# A mathematical model for the spread of COVID-19 and control mechanisms in Saudi Arabia

**DOI:** 10.1186/s13662-021-03410-z

**Published:** 2021-05-14

**Authors:** Mostafa Bachar, Mohamed A. Khamsi, Messaoud Bounkhel

**Affiliations:** 1 Department of Mathematics, College of Sciences, King Saud University, Riyadh, Kingdom of Saudi Arabia; 2 Department of Mathematics, Kalifa University, Abu Dhabia, United Arab Emirates

**Keywords:** Contact tracing, Testing, Quarantine, *COVID-19*EIISSRREx-model, Stability, Parameter estimations

## Abstract

In this work, we develop and analyze a nonautonomous mathematical model for the spread of the new corona-virus disease (*COVID-19*) in Saudi Arabia. The model includes eight time-dependent compartments: the dynamics of low-risk $S_{L}$ and high-risk $S_{M}$ susceptible individuals; the compartment of exposed individuals *E*; the compartment of infected individuals (divided into two compartments, namely those of infected undiagnosed individuals $I_{U}$ and the one consisting of infected diagnosed individuals $I_{D}$); the compartment of recovered undiagnosed individuals $R_{U}$, that of recovered diagnosed $R_{D}$ individuals, and the compartment of extinct *Ex* individuals. We investigate the persistence and the local stability including the reproduction number of the model, taking into account the control measures imposed by the authorities. We perform a parameter estimation over a short period of the total duration of the pandemic based on the *COVID-19* epidemiological data, including the number of infected, recovered, and extinct individuals, in different time episodes of the *COVID-19* spread.

## Introduction

In the last century several compartmental models in epidemiology are derivatives of the type Susceptible, Infected, and Recovered individuals (*SIR*), see for example [1, 2] for further details and recent modeling contributions with more references therein [3–7]. The most widely used models, and among the simplest ones, proved to be those proposed by Kermack and McKendrick [8–10]. One of the shortcomings of this work is the oversimplification of two different effects: *(i)* susceptible; and *(ii)* infectious, which are both lumped into a single effect. This kind of simplification does not take into account an individual’s degree of exposure to the infection. Hospital staff members or individuals with weakened immune systems or with chronic diseases, for example, should be placed in a category different from that of the individuals who, due to their occupation, age, or physical condition are less susceptible to contracting the infection. In particular, we quantitatively and qualitatively distinguish these two groups by considering the category of susceptible individuals as the union of two different compartments: that of higher-risk individuals and the compartment of lesser-risk individuals.

Extensions of *SIR*-type models have been proposed by several authors, see for example [11–14] with more reference therein, trying to capture the changes in the dynamics of the model interaction, particularly with respect to the reproductive number $\mathscr{R}_{0}$. An overview of some of the mathematical models in epidemiology which appeared in the literature is given in [15], enhanced with a recent publication on *COVID-19* modeling a series of the parameter values (rates) given in [3, 16–18]. Such parameters will provide good guidelines for our parameter estimation procedure.

Given the complexity of the biological systems and the interactions between given subsystems, where we have data limitations, mathematical modeling and analysis are needed to quantify such iterations and understand the behavior of such subsystems. The theoretical result such as the existence of positive solutions and their boundedness are important by nature, such as the number of individuals or the biomass dependent on the given model, where they should be positive and bounded. In addition to studying the stability of such a system of equations, the models must be as simple as possible and complex enough as necessary to reflect the reality, see for example [3, 19]. Consequently, real life data collection is very important for the parameter estimations in order to understand the interaction between the given subsystems. A broad overview of model validation is presented in [19–21], where more details about parameter estimations are given.

Recently, Lin et al. in [22] proposed a compartmental model with time-dependent transmission rate, which incorporates the impact of governmental action that could be modeled by step functions. On the other hand, Lin’s model should be in correspondence with the understanding of the quantity and quality of the available data. In addition to dividing the susceptible individual into higher- and lesser-risk compartments, we suggest splitting the compartment of infected individuals into the subcompartments consisting of diagnosed- and undiagnosed-infected individuals. This method will be helpful in controlling the outbreak of *COVID-19* (see the model proposed by Chowell et al. [23], which concerns the *SARS* outbreak in Ontario, Hon Kong, and Singapore, where they show that diagnosing and isolation help control the spread of the virus). Due to the large scale of the model, we conduct a careful sensitivity analysis to determine which model parameters have a strong influence on the dynamics of the system and therefore can be determined accurately in the parameter estimations, as was demonstrated in [19, 20].

In Sect. 2, we develop a nonautonomous mathematical model for the spread of the new coronavirus disease (*COVID-19*) in Saudi Arabia, taking into account the control measures imposed by the authorities, which are time dependent. The model includes eight time-dependent compartments: the dynamics of low-risk $S_{L}$ and high-risk $S_{M}$ susceptible individuals; the compartment of exposed individuals *E*; the compartment of infected individuals (divided into two compartments, namely that of infected undiagnosed individuals $I_{U}$ and the one consisting of infected diagnosed individuals $I_{D}$); the compartment of recovered undiagnosed individuals $R_{U}$, that of recovered diagnosed $R_{D}$ individuals, and the compartment of extinct *Ex* individuals. In Sect. 3, we investigate the qualitative aspect of the model including the model persistence and local stability, and Sect. 4 is devoted to the quantitative dynamics of the spread and the parameter estimation over a short period of the total duration of the pandemic based on the *COVID-19* data, including the number of infected, recovered, and extinct individuals in different episodes of the *COVID-19* spread. Finally, concluding remarks and future directions of our research are offered in Sect. 5.

## Model development

**Figure 1:**
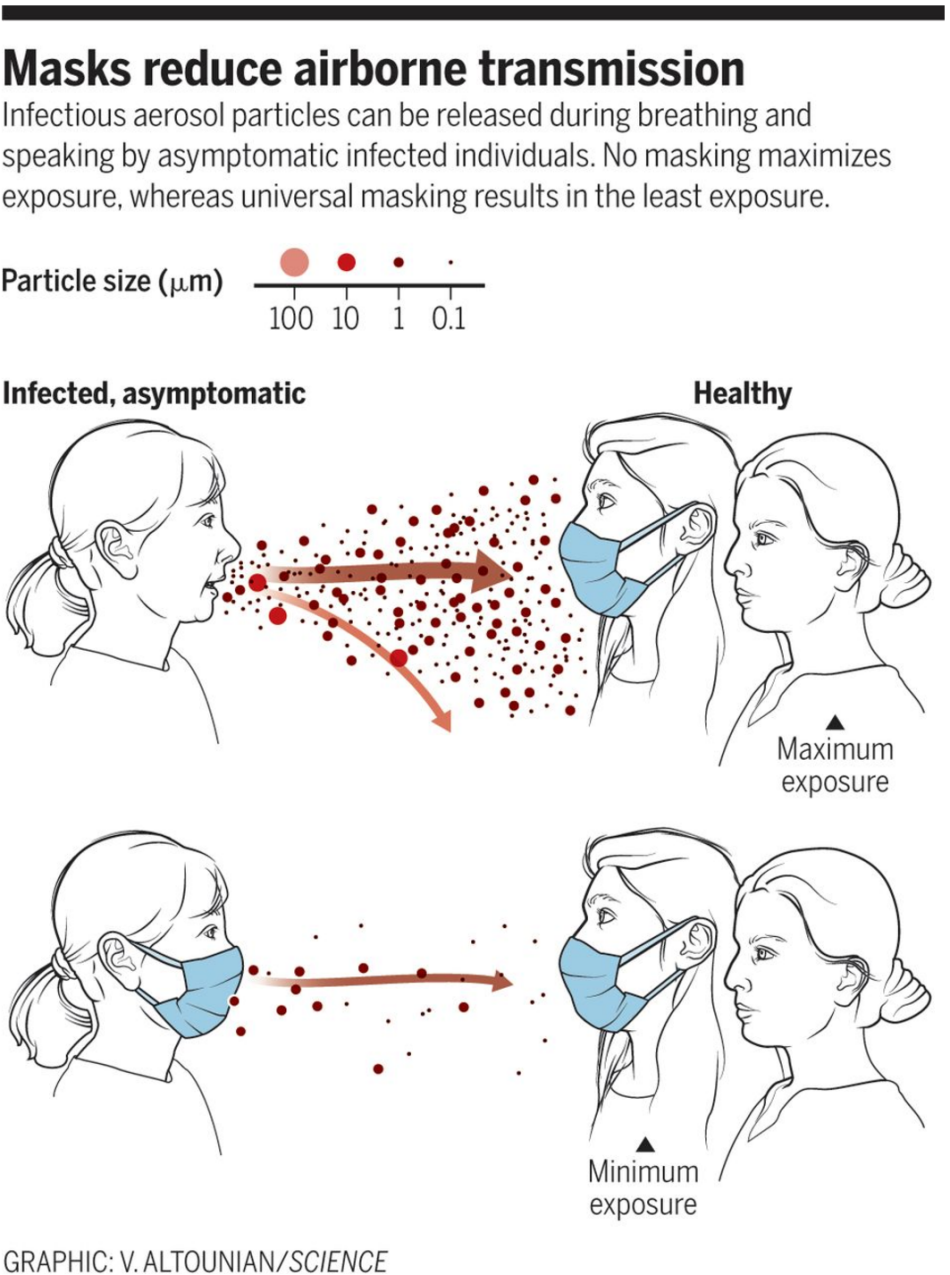
Graphic of V. Altounian [26], showing that masks reduce airborne transmission

**Figure 2:**
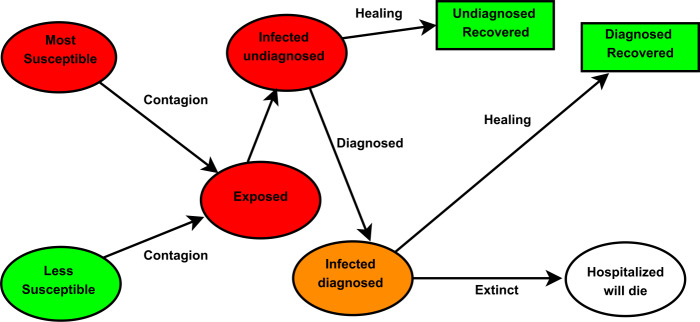
Diagram of the schematic representation of the flow of individuals among different stages of the infection. The mathematical model is called EIISSRREx-model and considers two distinct susceptible compartments: $S_{M}$ the most susceptible individuals and $S_{L}$ the less susceptible individuals. Also, there are uninfected *I*, infected individuals, asymptomatic, and symptomatic individuals. $I_{U}$ stands for the infected individuals and there are asymptomatic/undiagnosed (undetected) and those who will recover $R_{U}$; $R_{D}$ represents those diagnosed, hospitalized individuals that will recover; *E* represents the group of those diagnosed hospitalized individuals who die

As mentioned above, the compartment of susceptible individuals is divided into two further subcompartments: that consisting of the most susceptible individuals, denoted by $S_{M}$, with higher risk of contracting the *COVID-19* disease and the group of individuals with lower likelihood of getting infected. The latter will be called the compartment of less susceptible individuals and will be denoted by $S_{L}$. These groups of individuals will be considered according to how closely they follow the sanitary recommendations of the authorities in their daily life, namely, whether they often wash their hands, regularly disinfect their surroundings, and wear face masks. In general, susceptible individual are not infected by the disease-causing pathogen. These individuals are socially active and within certain limits follow the sanitary protection measures suggested by the US Center for Disease Control and Prevention *(CDC)*, such as social distancing of 1.5–2 m, regular hand washing, and wearing of face masks in their daily activities, in order to limit the airborne transmission of *COVID-19*, see [24]. Further measures might be needed for reducing airborne transmission of *COVID-19*, such as room ventilation and regular disinfection, which lead to effectively limiting the concentration of SARS-CoV-2 in aerosols, see [25]. In practice, however, most of the time social distancing is not respected. Yet, the sanitary protection provided by the universal wearing of masks represents a promising practice for reducing the transmission of the *COVID-19* infection, as was demonstrated in [26]. The epidemiological data provided at the beginning of the pandemic by a number countries, such as Taiwan, Japan, Hong Kong, Singapore, and South Korea, show the efficacy of universal masking as a control measure, even in the absence of a severe lockdown during the pandemic. In [26] the necessity of masking and testing in the fight against asymptomatic spread in aerosols and droplets is shown, and evidence is provided of the fact that no masking maximizes exposure, whereas the least level of exposure is achieved through universal masking. The universal use of face masks, it is shown in the work in point, helps reduce the size of the spread particles from 100 *μ*m to 1–0.1 *μ*m, especially in asymptomatic people and those with mild symptoms, see [27] and Fig. 1. Prather et al. [26] demonstrate that the aerosol filtering efficiency of different materials, thickness, and layers used in properly fitted homemade masks was similar to that of medical masks, see [28]. Tellier et al. [29] show that in still air, a 100 *μ*m droplet will drop to the ground from 2.4384 m in 4.6 s, whereas a 1 *μ*m aerosol particle will take 12.4 hours. The *(CDC)* recommends 1.8288 m for social distancing, but this recommendation might not be sufficient for indoor conditions, where aerosols can remain airborne and accumulate for hours, see [30, 31]. The authors in [31] show that breezes and winds often transport infected droplets and aerosols long distances. Further experimental research is needed in order to more precisely characterize the transport and to achieve a better understanding of the relevance of airborne transmission of the *COVID-19* infection [26]. For the simulation of *COVID-19*, the epidemiological EIISSRREx-model, for all $t\geq t_{0}$, would be formulated from Fig. 2 as follows:

1 $$\begin{aligned}& \begin{aligned}[b] \frac{dE(t)}{dt}& = \frac{S_{M}(t)+\mu _{L}(t)S_{L}(t)}{N} \bigl(C_{M}(t) \alpha _{E}E(t)+C_{M}(t)\alpha _{I_{U}}I_{U}(t) \\ & \quad {}+ C_{M}(t)\alpha _{I_{D}}I_{D}(t) \bigr)-\beta _{E}(t)E(t), \end{aligned} \end{aligned}$$

2 $$\begin{aligned}& \frac{dI_{U}(t)}{dt} = \beta _{E}(t)E(t)- \bigl(\beta _{I_{U}}(t)+ \beta _{R_{U}}(t) \bigr)I_{U}(t), \end{aligned}$$

3 $$\begin{aligned}& \frac{dI_{D}(t)}{dt} = \beta _{I_{U}}(t)I_{U}(t)- \bigl(\beta _{R_{D}}(t)+ \beta _{Ex}(t) \bigr)I_{D}(t), \end{aligned}$$

4 $$\begin{aligned}& \begin{aligned}[b] \frac{dS_{M}(t)}{dt} &= -\frac{S_{M}(t)}{N} \bigl(C_{M}(t)\alpha _{E}E(t)+C_{M}(t) \alpha _{I_{U}}I_{U}(t) \\ & \quad {}+ C_{M}(t)\alpha _{I_{D}}I_{D}(t) \bigr), \end{aligned} \end{aligned}$$

5 $$\begin{aligned}& \begin{aligned} \frac{dS_{L}(t)}{dt}& = -\mu _{L}(t)\frac{S_{L}(t)}{N} \bigl(C_{M}(t) \alpha _{E}E(t)+C_{M}(t)\alpha _{I_{U}}I_{U}(t) \\ & \quad {}+ C_{M}(t)\alpha _{I_{D}}I_{D}(t) \bigr), \end{aligned} \end{aligned}$$

6 $$\begin{aligned}& \frac{dR_{U}(t)}{dt} = \beta _{R_{U}}(t)I_{U}(t), \end{aligned}$$

7 $$\begin{aligned}& \frac{dR_{D}(t)}{dt} = \beta _{R_{D}}(t)I_{D}(t), \end{aligned}$$

8 $$\begin{aligned}& \frac{dEx(t)}{dt} = \beta _{Ex}(t)I_{D}(t). \end{aligned}$$

Here, *E* represents the number of individuals in the incubation period, that is, the number of persons exposed to the virus with no visible symptoms. $I_{U}$ represents the number of infected, undiagnosed individuals, asymptomatic, and symptomatic individuals with mild symptoms that have not been identified by the authorities. $I_{D}$ represents the number of infected, diagnosed individuals, that is, the number of persons that have been officially identified as infected and are either hospitalized or in quarantine at home. Those individuals are registered in an official applications provided by the local Saudi Authority, called “Tawakkalna ()”, see “https://ta.sdaia.gov.sa/en/index”. Such application will help with contact tracing. Therefore as we increase the number of testing, we will be able to localize clustering of infected individuals which will help to reduce their numbers. $S_{M}$ represents the number of most susceptible individuals, with higher risk of contracting the *COVID-19* infection. $S_{L}$ represents the number of individuals that are less susceptible to the *COVID-19* infection. $R_{U}$ represents the number of recovered, undiagnosed individuals, who are not being officially identified as such. $R_{D}$ represents the number of infected, recovered individuals officially identified as such and finally *Ex* represents the number of individuals who are deceased from *COVID-19* (called extinct individuals). The parameter $\mu_{L}$ represents the reduction risk factor of the *COVID-19* infection for the less susceptible individuals $S_{L}$. $C_{M}(t)$ represents the efficiency of the control measures imposed by the authorities, such as the lockdowns. This quantity can be modeled as a step function in the following fashion:

9 $$\begin{aligned} C_{M}(t) = & \sum^{n}_{i=0}\chi _{[t_{i},t_{i+1}]}(t)\kappa _{i}, \end{aligned}$$

where

$$\begin{aligned} {\chi }_{[t_{i},t_{i+1}]}(t) = \textstyle\begin{cases} 1, &\text{if $t_{i}\leq t < t_{i+1}$, $i=0,\dots ,n$}, \\ 0, &\text{otherwise}. \end{cases}\displaystyle \end{aligned}$$

The time episode of the lockdown imposed by the authorities is denoted by an increasing positive sequence $(t_{i})_{0\leq i \leq n+1}$, and the authorities-controlled parameters are given by $\kappa _{i} \in (0,1)$, $i=0,\ldots ,n$. The positive initial conditions are given by $S_{M}(t_{0})=S^{0}_{M}=\rho N$ and $S_{L}(t_{0})=S_{L}^{0}=(1-\rho ) N$, where $t_{0}\geq 0$ represents any initial time and *ρ* is the proportion of the population size *N* that is initially at higher risk of contracting the infection, see [23]. The disease constant rates $\alpha _{E}, \alpha _{I},\alpha _{I_{D}},\alpha _{I_{U}} \in (day^{-1})$ of individuals in the corresponding compartment are supposed to be constant, without taking into account the control measures. Without loosing any generality, we can suppose that all parameters are piecewise constants, as in (9), which means

$$\begin{aligned} \theta (t) = & \sum^{n}_{i=0}\chi _{[t_{i},t_{i+1}]}(t)\theta _{i}, \end{aligned}$$

where

$$ \theta (t)= \bigl(\alpha _{E}(t),\alpha _{I_{U}}(t),\alpha _{I_{D}}(t), \beta _{E}(t),\beta _{I_{U}}(t),\beta _{R_{U}}(t),\beta _{R_{D}}(t), \beta _{Ex}(t) \bigr) $$

represents the time-dependent model parameters, and

$$ \theta _{i}= ({\alpha _{E}}_{i},{\alpha _{I_{U}}}_{i},{\alpha _{I_{D}}}_{i},{ \beta _{E}}_{i},{\beta _{I_{U}}}_{i},{\beta _{R_{U}}}_{i},{\beta _{R_{D}}}_{i},{ \beta _{Ex}}_{i} ),\quad i=0,\dots ,n, $$

are the model parameters constant over each time episode of the lockdown at the interval $[t_{i},t_{i+1}]$, $0\leq i \leq n$. Table 1 displays a detailed description of the parameters (including their units).

**Table 1:** Parameters used in model (1–8)

Parameters	Meaning	Values	Ref.
$\alpha _{E}$	Contact disease rate of a person in compartment *E* at time *t*	Estimated $day^{-1}$	Table 2
$\alpha _{I_{U}}$	Contact disease rate of a person in compartment ${I}_{U}$ at time *t*	Estimated $day^{-1}$	Table 2
$\alpha _{I_{D}}$	Contact disease rate of a person in compartment ${I}_{Diag}$ at time *t*	Estimated $day^{-1}$	Table 2
$\beta _{E}$	Transition rate of a person in compartment *E* at time *t*	Estimated $day^{-1}$	Table 2
$\beta _{I_{U}}$	Transition rate of a person in compartment ${I}_{U}$ at time *t*	Estimated $day^{-1}$	Table 2
$\beta _{R_{U}}$	Rate at which an undiagnosed infected person recovers at time *t*	Estimated $day^{-1}$	Table 2
$\beta _{R_{D}}$	Rate at which a diagnosed infected person recovers at time *t*	Estimated $day^{-1}$	Table 2
$\beta _{Ex}$	Rate at which a diagnosed infected person dies at time *t*	Estimated $day^{-1}$	Table 2
$\mu _{L}$	Reduction risk factor of infection in compartment $S_{L}$ at time *t*	—	Table 2
*ρ*	Proportion of the population size *N* that is initially at higher risk of contracting the infection	0.4	[23]
*N*	Total size of the population	30⋅10^6^	—

The positive initial conditions $E(t_{0})=E^{0}$, $I_{D}(t_{0})=I_{D}^{0}$, and $I_{U}(t_{0})=I_{U}^{0}$ are given in $[0,\infty )$. The transition rate $\beta _{E}$ from the exposed compartment *E* to the infected compartment *I* is supposed to be constant at the beginning of the pandemic according to [32].

## Model analysis

The model provides a description of the mass transfer property between the compartments. It follows that each solution of the equations involved in it (1)–(8) should be positive, bounded, and persistent. The sum of the states (total population) is constant and equal to *N*, i.e.,

$$ E(t)+I_{U}(t)+I_{D}(t)+S_{M}(t)+S_{L}(t)+R_{U}(t)+R_{D}(t)+Ex(t)=N. $$

In the sequel, we set $C(\mathbb{R}^{+},\mathbb{R}^{8})$ to denote the space of all continuous functions from ${\mathbb{R}}^{+}$ into ${\mathbb{R}}^{8}$. It is a well-established mathematical fact [33] that the autonomous system of differential equations (4)–(8), which depends on the initial conditions $E(t_{0})= E^{0}$, $I_{U}(t_{0})=0$, $I_{D}(t_{0})=0$, $S_{M}(t_{0})=S_{M}^{0}$, $S_{L}(t_{0})=S_{L}^{0}$, $R_{U}(t_{0}) =0$, $R_{D}(t_{0}) = 0$, $Ex(t_{0}) =0$, is uniquely solvable.

### Theorem 3.1

*There exists a unique solution to the system of equations* (1)*–*(8) *in* $C(\mathbb{R}^{+},\mathbb{R}^{8})$. *Furthermore*, *the solution is positive and bounded from above*.

### Proof

The right-hand side of each of equations (1)–(8) is continuously differentiable almost everywhere in $C(\mathbb{R}^{+},\mathbb{R}^{8})$. We refer the reader to [33] for the mathematical result that guarantees that under such circumstances the system of equations (1)–(8) with the given initial conditions is uniquely solvable for all $t \geq 0$.

Denote the solution of (4) by $S_{M}(t)$. If for any

$$ \tau _{0}=\inf \bigl\{ t>0, \text{ where }S_{M}(t) < 0\bigr\} \quad \text{held that } S_{M}( \tau _{0})=0, $$

then we would have $\frac{dS_{M}(t)}{dt}_{|{\tau _{0}}}<0$. From equation (4), we get

$$\begin{aligned} 0 >&\frac{dS_{M}(t)}{dt}_{|{\tau _{0}}}\\ = & - \frac{S_{M}(\tau _{0})}{N} \bigl(C_{M}(\tau _{0})\alpha _{E}E(\tau _{0})+C_{M}(t) \alpha _{I_{U}}I_{U}(\tau _{0})+\cdots +C_{M}(t)\alpha _{I_{D}}I_{D}(\tau _{0}) \bigr) \\ =&0, \end{aligned}$$

which is a contradiction. Therefore, $S_{M}(t)\geq 0$ for all $t \geq t_{0}$. In a similar way, we can prove that $S_{L}(t)$ is positive for all $t\geq 0$.

Denote the solution of equation (1) by $E(t)$. Assuming the existence of $\tau _{1}$ such that

$$ \tau _{1}=\inf \bigl\{ t>0, \text{ where }E(t) < 0\bigr\} , $$

such that $E(\tau _{1})=0$, we would have $\frac{dE(t)}{dt}_{|{\tau _{1}}}<0$. From equation (1), we have

$$ \mathcal{G}(t)=C_{M}(t)\alpha _{E}E(t)+C_{M}(t) \alpha _{I_{U}}I_{U}(t)+C_{M}(t) \alpha _{I_{D}}I_{D}(t), $$

and it follows that

10 $$\begin{aligned} \frac{dE(t)}{dt}_{|{\tau _{1}}} = & \frac{S_{M}(\tau _{1})+\mu _{L}(\tau _{1})S_{L}(\tau _{1})}{N} \mathcal{G}(\tau _{1}) . \end{aligned}$$

Using equations (2) and (3), we obtain

$$\begin{aligned} \frac{d}{dt} \begin{pmatrix} I_{U}(t) \\ I_{D}(t) \end{pmatrix}_{|{\tau _{1}}} = & \begin{pmatrix} - (\beta _{I_{U}}(\tau _{1})+\beta _{R_{U}}(\tau _{1}) )&0 \\ \beta _{I_{U}}(\tau _{1})&- (\beta _{R_{D}}(\tau _{1})+\beta _{Ex}( \tau _{1}) ) \end{pmatrix} \begin{pmatrix} I_{U}(\tau _{1}) \\ I_{D}(\tau _{1}) \end{pmatrix}. \end{aligned}$$

Then we have

$$ \begin{pmatrix} I_{U}(\tau _{1}) \\ I_{D}(\tau _{1}) \end{pmatrix}=\Omega (\tau _{1},t_{0}) \begin{pmatrix} I_{U}(t_{0}) \\ I_{D}(t_{0}) \end{pmatrix}, $$

where

$$ \Omega (\tau _{1},t_{0})= \begin{pmatrix} e^{- \int ^{\tau _{1}}_{t_{0}} (\beta _{I_{U}}(s)+ \beta _{R_{U}}(s) )\,ds}&0 \\ \mathcal{A}(\tau _{1},t_{0})&e^{- \int ^{\tau _{1}}_{t_{0}} (\beta _{R_{D}}(s)+\beta _{Ex}(s) )\,ds} \end{pmatrix} $$

and

$$ \mathcal{A}(\tau _{1},t_{0})= \int ^{\tau _{1}}_{t_{0}} \beta _{I_{U}}(\sigma )e^{- \int ^{\sigma }_{t_{0}} ( \beta _{I_{U}}(s)+\beta _{R_{U}}(s) )\,ds}e^{- \int ^{ \tau _{1}}_{\sigma } (\beta _{R_{D}}(s)+\beta _{Ex}(s) )\,ds}\,d \sigma . $$

Then, for any positive initial condition, $I_{U}(\tau _{1})$ and $I_{D}(\tau _{1})$ are positive. Again, equation (10) yields

$$\begin{aligned} 0>\frac{dE(t)}{dt}_{|{\tau _{1}}} = & \frac{S_{M}(\tau _{1})+\mu _{L}(\tau _{1})S_{L}(\tau _{1})}{N} \mathcal{G}(\tau _{1})\geq 0. \end{aligned}$$

This is a contradiction and thus it must hold that $E(t)\geq 0$ for all $t \geq t_{0}$.

It can be shown analogously that $I_{U}(t)$, $I_{D}(t)$, $R_{U}(t)$, $R_{D}(t)$, and $Ex(t)$ are positive for all $t\geq 0$, hence the positiveness of the solution of system (1)–(8).

Next, we show the boundedness of $S_{M}(t)$, $S_{L}(t)$, $E(t)$, $I_{U}(t)$, $I_{D}(t)$, $R_{U}(t)$, $R_{D}(t)$, and $Ex(t)$ for all $t>0$. We tackle first the function $S_{M}(t)$. Equation (4) yields, for $t\geq t_{0}$,

11 $$\begin{aligned} S_{M}(t) = & e^{- \int ^{t}_{t_{0}} \frac{\mathcal{G}(s)}{N}\,ds}S_{M}^{0}\leq S_{M}^{0}, \end{aligned}$$

and from equation (5)

12 $$\begin{aligned} S_{L}(t) = & e^{- \int ^{t}_{t_{0}}\mu _{L} \frac{\mathcal{G}(s)}{N}\,ds}S_{L}^{0}\leq S_{L}^{0}. \end{aligned}$$

Thus, $S_{M}(t)$ and $S_{L}(t)$ are bounded from above.

Now, we show that any given solution of (1)–(8) is bounded. It is clear from the model equations (4)–(8) that we have

$$ E(t)+I_{U}(t)+I_{D}(t)+R_{U}(t)+R_{D}(t)+Ex(t) \leq N, $$

and by using the positivity of each state equation, we can conclude that, for all $t\geq 0$, all solutions are bounded from above. □

Throughout this paper, we use the following notations for the lim sup and the lim inf of output of the model in each compartment:

$$ \overline{X} = \limsup_{t\rightarrow \infty } X(t) ,\qquad \underline{X}= \liminf _{t\rightarrow \infty } X(t). $$

### Theorem 3.2

*Model* (1)*–*(8) *is persistent*. *This is to say that the components of each solution are eventually uniformly bounded from above and away from zero*.

### Proof

Since $S_{M}^{0}$ and $S_{L}^{0}$ are a finite, using the positivity and boundedness of the operator $C_{M}(t)\alpha _{E}E(t)+C_{M}(t)\alpha _{I_{U}}I_{U}(t)+C_{M}(t) \alpha _{I_{D}}I_{D}(t)$ in integrals (11) and (12), we conclude that

$$ 0< \underline{S_{M}}< \overline{S_{M}}< \infty \quad \text{and}\quad 0< \underline{S_{L}}< \overline{S_{L}}< \infty . $$

Again, since *E*, $I_{U}$, and $I_{D}$ are positive and bounded, and from equations (2)–(3) and (6)–(8), we have

$$\begin{aligned}& I_{U}(t) = \int _{t_{0}}^{t}e^{- \int ^{t}_{s} ( \beta _{I_{U}}(\sigma )+\beta _{R_{U}}(\sigma ) )\,d\sigma }\beta _{E} E(s)\,ds, \\& I_{D}(t) = \int _{t_{0}}^{t}e^{- \int ^{t}_{s} ( \beta _{R_{D}}(\sigma )+\beta _{Ex}(\sigma ) )\,d\sigma }\beta _{I_{U}} I_{D}(s)\,ds, \\& R_{U}(t) = \int _{t_{0}}^{t}\beta _{R_{U}} (s)I_{U}(s)\,ds, \\& R_{D}(t) = \int _{t_{0}}^{t}\beta _{R_{D}}(s) I_{D}(s)\,ds, \\& Ex(t) = \int _{t_{0}}^{t}\beta _{Ex}(s)I_{D}(s)\,ds. \end{aligned}$$

Then

$$\begin{aligned}& 0< \underline{I_{U}}< \overline{I_{U}}< \infty ,\qquad 0< \underline{I_{D}}< \overline{I_{D}}< \infty , \\& 0< \underline{R_{U}}< \overline{R_{U}}< \infty ,\qquad 0< \underline{R_{D}}< \overline{R_{D}}< \infty , \quad \text{and}\quad 0< \underline{Ex}< \overline{Ex}< \infty . \end{aligned}$$

Now, we will show that $\underline{E}=\liminf_{t\rightarrow \infty } E(t)>0$. Suppose that $\underline{E}<\overline{E}$. Then, by using the fluctuation lemma, see for example Hirsch [34], there exists a sequence $\{t_{k}\}_{k=1}^{\infty }$ such that, for all $k\geq 1$,

$$\begin{aligned} \frac{dE(t)}{dt}_{|{t_{k}}} = & 0, \qquad \lim_{k\rightarrow \infty }E(t_{k})= \underline{E}. \end{aligned}$$

It is clear from the model equation (1) that we have

$$\begin{aligned} 0 = & \frac{S_{M}(t_{k})+\mu _{L}S_{L}(t_{k})}{N} \bigl(C_{M}(t_{k}) \alpha _{E}E(t_{k})+C_{M}(t_{k})\alpha _{I_{U}}I_{U}(t_{k}) \\ &{}+ C_{M}(t_{k})\alpha _{I_{D}}I_{D}(t_{k}) \bigr)-\beta _{E}(t_{k})E(t_{k}), \end{aligned}$$

then

$$\begin{aligned} 0 \geq & \frac{m_{S_{M}}+m_{\mu _{L}}m_{S_{L}}}{N} \bigl(m_{C_{M}} \alpha _{E}E(t_{k})+m_{C_{M}} \alpha _{I_{U}}m_{I_{U}} \\ &{}+ m_{C_{M}}\alpha _{I_{D}}m_{I_{D}(t_{k})} \bigr)-\beta _{E}(t_{k})E(t_{k}), \end{aligned}$$

where $m_{\Xi }=\min_{t\in [t_{0},\infty )}\Xi (t)>0$, $\Xi :=C_{M}, S_{M},S_{L},I_{U},I_{D}, \mu _{L}$, and $\overline{\beta _{E}}=\max_{t\in [t_{0},\infty )} \beta _{E}(t) $. Therefore, as $k\rightarrow \infty $, we obtain

$$\begin{aligned} 0 \geq & \frac{m_{S_{M}}+m_{\mu _{L}}m_{S_{L}}}{N} (m_{C_{M}} \alpha _{E} \underline{E}+m_{C_{M}}\alpha _{I_{U}}m_{I_{U}}) \\ &{}+ m_{C_{M}}\alpha _{I_{D}}m_{I_{D}} )-\overline{\beta _{E}} \underline{E}. \end{aligned}$$

If we have $\underline{E}=0$, then we get a contradiction as we have

$$\begin{aligned} 0 \geq & \frac{m_{S_{M}}+m_{\mu _{L}}m_{S_{L}}}{N} (m_{C_{M}} \alpha _{I_{U}}m_{I_{U}}+m_{C_{M}} \alpha _{I_{D}}m_{I_{D}} )>0. \end{aligned}$$

If we have $\lim_{t\rightarrow \infty }{E}=0$, the model equation (1) implies that $\frac{dE(t)}{dt}>\gamma >0$ as $t\rightarrow \infty $, and we have $\lim_{t\rightarrow \infty }{E}=\infty $, which will be a contradiction. Finally, if we suppose $\underline{E}=\overline{E}$, then $\lim_{t\rightarrow \infty }{E}$ exists and *E* is eventually uniformly bounded from above and away from zero, which is the desired conclusion. □

It is clear that the last three equations (6)–(8) can be solved explicitly and that the solutions will depend on the infected compartments (6)–(7). We denote the susceptible compartment by $\mathbf{x}_{S}= (S_{M},S_{L} )^{T}$ and the infected compartment by $\mathbf{x}_{I}= (E,I_{U},I_{D} )^{T}$. Then the model equations (1)–(5), can be written as a feedback control structure

13 $$\begin{aligned}& \frac{d \mathbf{x}_{I}(t)}{dt} = \mathbf{A}(t)\mathbf{x_{I}}(t)+ \mathbf{b} u(t), \end{aligned}$$

14 $$\begin{aligned}& \frac{d \mathbf{x}_{S}(t)}{dt} = -x_{S}(t)\mathbf{D}(t)\mathbf{x}_{S}(t), \end{aligned}$$

15 $$\begin{aligned}& x_{S}(t) = \frac{C_{M}(t)}{N} (\alpha _{E},\alpha _{I_{U}}, \alpha _{I_{D}} )\mathbf{x_{I}}(t), \end{aligned}$$

16 $$\begin{aligned}& u(t) = \bigl(S_{M}(t)+\mu _{L}(t)S_{L}(t) \bigr)x_{S}(t), \end{aligned}$$

and the remaining recovered and extinct compartments (8)–(6) can be denoted as $\mathbf{x}_{REx}= (R_{U},R_{D},Ex )^{T}$, which depend on the infected compartment $\mathbf{x}_{I}$ as follows:

$$ \frac{d \mathbf{x_{REx}}}{dt}(t)=\mathbf{B}(t)\mathbf{x}_{I}(t), $$

where

$$\begin{aligned}& \mathbf{A}(t)= \begin{pmatrix} -\beta _{E}(t)&0&0 \\ \beta _{E}(t)&- (\beta _{I_{U}}(t)+\beta _{R_{U}}(t) )&0 \\ 0&\beta _{I_{U}}(t)&- (\beta _{R_{D}}(t)+\beta _{Ex}(t) ) \end{pmatrix},\qquad \mathbf{b}= \begin{pmatrix} 1 \\ 0 \\ 0 \end{pmatrix}, \\& \mathbf{B}(t)= \begin{pmatrix} 0&\beta _{I_{U}}(t)&0 \\ 0&0&\beta _{I_{D}}(t) \\ 0&0&\beta _{Ex}(t) \end{pmatrix}, \quad \text{and}\quad \mathbf{D}(t)= \begin{pmatrix} 1&0 \\ 0&\mu _{L}(t) \end{pmatrix}. \end{aligned}$$

To find the basic reproduction number $\mathscr{R}_{0}$, the average number of individuals that one single infected individual is capable of infecting during each time episode of the lockdown in the interval $[t_{i},t_{i+1}]$, $0\leq i \leq n$, should be kept in mind. In other words, we are going to protect uninfected individuals as we have no control measure. In this case, the number of uninfected individuals will depend on the size of the population and not on the time period of the spread. Therefore, to study the stability, and for the sake of simplicity, we will suppose that all parameters are constant. The possible equilibrium of the system of equations (1)–(8) is given by

$$\begin{aligned}& \overline{E}=0,\qquad \overline{I}_{U}=0,\qquad \overline{I}_{D}=0,\qquad \overline{S}_{M} \geq 0,\qquad \overline{S}_{L}\geq 0,\\& \overline{R}_{U}\geq 0,\qquad \overline{R}_{Diag} \geq 0,\qquad \overline{Ex} \geq 0 \end{aligned}$$

with

$$ \overline{S}_{M}+ \overline{S}_{L}+\overline{R}_{U}+ \overline{R}_{D}+ \overline{Ex}=N. $$

Therefore only most susceptible, low susceptible, received, and deceased individuals are present, which means that the epidemic is over. It is clear that the rate of appearance $\mathscr{F}=(\mathscr{F}_{i})_{1\leq i\leq 8}$ of new infections in each compartment *i* and the rate of transfer $\mathscr{V}^{+}=(\mathscr{V}^{+}_{i})_{1\leq i\leq 8}$ (resp. $\mathscr{V}^{+}=(\mathscr{V}^{-}_{i})_{1\leq i\leq 8}$ of individuals into (resp. out of) in each compartment *i* of the model equations can be written as follows:

$$\begin{aligned}& \mathscr{F}(\mathbf{x})= \biggl(\frac{S_{M}+\mu _{L}S_{L}}{N} (\alpha _{E}E+ \alpha _{I_{U}}I_{U}+\alpha _{I_{D}}I_{D}), \mathbf{0}_{1\times 7} \biggr)^{T}, \\& \mathscr{V}^{+}(\mathbf{x})= (0,\beta _{E}E,\beta _{I_{U}}I_{U},0,0, \beta _{R_{U}}I_{U},\beta _{R_{D}}I_{U},\beta _{Ex}I_{D} )^{T}, \end{aligned}$$

and

$$\begin{aligned} \mathscr{V}^{-}(\mathbf{x}) = & \biggl(\beta _{E}E,(\beta _{I_{U}}+ \beta _{R_{U}})I_{U},(\beta _{R_{D}}+ \beta _{Ex})I_{D}, \frac{S_{L}}{N} (\alpha _{E}E+\alpha _{I_{U}}I_{U} \\ &{}+ \alpha _{I_{D}}I_{D}), \mu _{L} \frac{S_{L}}{N} (\alpha _{E}E+ \alpha _{I_{U}}I_{U}+ \alpha _{I_{D}}I_{D}),\mathbf{0}_{1\times 3} \biggr)^{T}, \end{aligned}$$

where $\mathbf{x}= (E,I_{U},I_{D},S_{M},S_{L},R_{U},R_{D},Ex )^{T}$. The model equations given in (1)–(8) can be rewritten as the following system of equations:

$$\begin{aligned} \frac{d \mathbf{x}}{dt}(t) = & \mathscr{F}(\mathbf{x})-\mathscr{V}( \mathbf{x}), \end{aligned}$$

where $\mathscr{V}=\mathscr{V}^{-}-\mathscr{V}^{+}$. To discuss the local asymptotic stability of the endemic steady state $\mathbf{X}^{*} = (0, 0,0, \overline{S}_{M}, \overline{S}_{L}, \overline{R}_{U}, \overline{R}_{D},\overline{Ex} )^{T}$, we linearize around the equilibrium $\mathbf{X}^{*}$. Using the notation for the corresponding linearized model around $\mathbf{X}= (E^{l},I_{U}^{l},I_{D}^{l},S_{M}^{l},S_{L}^{l},R_{U}^{l},R_{D}^{l},Ex^{l} )^{T}$, we have then

$$\begin{aligned} \frac{d \mathbf{X}}{dt}(t) = & \mathcal{J}\mathbf{X}(t), \end{aligned}$$

where

$$ \mathcal{J}= \begin{pmatrix} \alpha _{E} \frac{\overline{S}_{M}+\mu _{L}\overline{S}_{L}}{N}- \beta _{E}&\alpha _{I_{U}} \frac{\overline{S}_{M}+\mu _{L}\overline{S}_{L}}{N}&\alpha _{I_{D}} \frac{\overline{S}_{M}+\mu _{L}\overline{S}_{L}}{N}&\mathbf{0}_{1 \times 5} \\ \beta _{E}&- (\beta _{I_{U}}+\beta _{R_{U}} )&0&\mathbf{0}_{1 \times 5} \\ 0&\beta _{I_{U}}&- (\beta _{R_{D}}+\beta _{Ex} )&\mathbf{0}_{1 \times 5} \\ -\alpha _{E} \frac{\overline{S}_{M}}{N}&-\alpha _{I_{U}} \frac{\overline{S}_{M}}{N}&-\alpha _{I_{D}} \frac{\overline{S}_{M}}{N}&\mathbf{0}_{1\times 5} \\ -\mu _{L}\alpha _{E} \frac{\overline{S}_{M}}{N}&-\alpha _{I_{U}} \frac{\overline{S}_{M}}{N}&-\alpha _{I_{D}} \frac{\overline{S}_{M}}{N}&\mathbf{0}_{1\times 5} \\ 0&\beta _{R_{U}}&0&\mathbf{0}_{1\times 5} \\ 0&0&\beta _{R_{D}}&\mathbf{0}_{1\times 5} \\ 0&0&\beta _{Ex}&\mathbf{0}_{1\times 5} \end{pmatrix}. $$

To understand the qualitative behavior of the model equations (1)–(8), we observe that the infected compartments *E*, $I_{U}$, $I_{D}$ in the equilibrium are zero and that the remaining compartments $S_{M}$, $S_{L}$, $R_{U}$, $R_{D}$, *Ex* are at the given equilibrium $\overline{S}_{M}$, $\overline{S}_{L}$, $\overline{R}_{U}$, $\overline{R}_{D}$, $\overline{Ex}$.

Now, if we set $\mathscr{F}(\mathbf{\widehat{x}})=0$ for all $\hat{x}={\left(E,I_{U},I_{D},S_{M},S_{L}\right)}^{T}=(\begin{array}{c} x_{I}\\ x_{S} \end{array})$, where $\mathbf{\widehat{x}}$ includes the infected subjects with susceptible individuals for the infection, it is clear that the new Jacobian matrix $\mathcal{\widehat{J}}$ has three negative eigenvalues given by $-\beta _{E}$, $-(\beta _{I_{U}}+\beta _{R_{U}})$, $-(\beta _{R_{D}}+ \beta _{Ex})$ and that the characteristic polynomial can be written as

$$\begin{aligned} \Delta (\xi ) = & \det (\xi I -\mathcal{\widehat{J}}), \\ = & \xi ^{3}+a_{2}\xi ^{2}+a_{1} \xi +a_{0}, \end{aligned}$$

where

$$\begin{aligned} a_{2} =&\beta _{E}+\beta _{R_{D}}+\beta _{I_{U}}+\beta _{R_{U}}+ \beta _{Ex}, \\ a_{1} =&(\beta _{R_{D}}+\beta _{Ex}) (\beta _{E}+\beta _{I_{U}}+\beta _{R_{U}})+ \beta _{E}(\beta _{I_{U}}+\beta _{R_{U}}), \\ a_{0} =&\beta _{E}(\beta _{I_{U}}+\beta _{R_{U}}) (\beta _{R_{D}}+ \beta _{Ex}). \end{aligned}$$

In the proof of the local asymptotic stability, we borrow the powerful tools from the theory of *M*-matrices [35–39]. For the reader’s convenience, we state the characterization of *M*-matrices with regard to the spectral radius (*SR*) and to the spectral abscissa (*SA*).

Recall that given a matrix *A*, its spectral radius, denoted by $\rho (A)$, is given by

17 $$\begin{aligned} \rho (A) = & \max \bigl\{ \vert \lambda \vert : \det (\lambda I-A)\bigr\} . \end{aligned}$$

(*SR*)[38, 39]; If $A \in R^{n\times n}$ can be written in the form $A=sI-B$, where $s>0$ and $B=(b_{ij})_{1\leq i,j\leq n}$ is nonnegative ($B\geq 0$, if $b_{ij}\geq 0$, $1\leq i$, $j\leq n$) and $s\geq \rho (B)$, then *A* is called an *M*-matrix.
(*SA*)[38, 39]; A matrix *A* is said to be an *M*-matrix if $A \in R^{n\times n}$ has the $Z^{n \times n}$ pattern, that is, if *A* belongs to the class
  $$ Z^{n \times n}=\bigl\{ A=(a_{ij})\in R^{n\times n}: a_{ij}\leq 0, i\neq j\bigr\} , $$
  and if each eigenvalue $\lambda _{i}(A)$ of *A* satisfies
  $$\begin{aligned} \Re \bigl(\lambda _{i}(A)\bigr) \geq & 0, \quad i=1,\dots ,n . \end{aligned}$$

Clearly, an *M*-matrix *A* is nonsingular if and only if the following condition holds: $s=\rho (B)$ for some $B\geq 0$ in (*SR*), so that $\Re (\lambda _{i}(A))> 0$ in (*SA*).

The following lemma will be useful throughout the characterization of the reproduction number $\mathscr{R}_{0}$. We refer the reader to [39, p. 159] and [40, p. 127] for further details.

### Lemma 3.3

([39, 40])

*Let* *V* *be a nonsingular* *M*-*matrix such that both* *J* *and* $JV^{-1}$ *have the* $Z^{n\times n}$ *sign pattern*. *Then* *J* *is a nonsingular* *M*-*matrix if and only if* $JV^{-1}$ *is a nonsingular* *M*-*matrix*.

Now, conditions $(A_{1})$–$(A_{5})$ in [41] are satisfied for the model equations (13)–(16). Therefore the rate of appearance of new infections in each compartment *i*, $\mathscr{\widehat{F}}=(\mathscr{\widehat{F}}_{i})_{1\leq i\leq 5}$, and the rate of transfer of individuals into (resp. out of) each compartment *i*, $\mathscr{\widehat{V}}^{+}=(\mathscr{\widehat{V}}^{+}_{i})_{1\leq i \leq 5}$ (resp. $\mathscr{\widehat{V}}^{+}=(\mathscr{\widehat{V}}^{-}_{i})_{1\leq i \leq 5}$, of the model equations (13)–(16) satisfy

$$\begin{aligned} \frac{d \mathbf{\widehat{x}}}{dt}(t) = & \mathscr{\widehat{F}}( \mathbf{\widehat{x}})- \mathscr{\widehat{V}}(\mathbf{\widehat{x}}), \end{aligned}$$

where

$$\begin{aligned}& \mathscr{\widehat{F}}(\mathbf{\widehat{x}})= \biggl( \frac{S_{M}+\mu _{L}S_{L}}{N} (\alpha _{E}E+\alpha _{I_{U}}I_{U}+ \alpha _{I_{D}}I_{D}),\mathbf{0}_{1\times 4} \biggr)^{T}, \\& \mathscr{\widehat{V}}^{+}(\mathbf{\widehat{x}})= (0,\beta _{E}E, \beta _{I_{U}}I_{U},0,0 )^{T}, \\& \begin{aligned} \mathscr{\widehat{V}}^{-}(\mathbf{\widehat{x}})& = \biggl(\beta _{E}E,( \beta _{I_{U}}+\beta _{R_{U}})I_{U},( \beta _{R_{D}}+\beta _{Ex})I_{D}, \frac{S_{M}}{N} ( \alpha _{E}E+\alpha _{I_{U}}I_{U} \\ & \quad {}+ \alpha _{I_{D}}I_{D}),\mu _{L} \frac{S_{L}}{N} (\alpha _{E}E+ \alpha _{I_{U}}I_{U}+ \alpha _{I_{D}}I_{D}) \biggr)^{T}, \end{aligned} \end{aligned}$$

and

$$\begin{aligned}& \begin{aligned} \mathscr{\widehat{V}}(\mathbf{\widehat{x}})& = \mathscr{\widehat{V}}^{-}( \mathbf{\widehat{x}})-\mathscr{\widehat{V}}^{+}( \mathbf{\widehat{x}}), \\ & = \biggl(\beta _{E}E,(\beta _{I_{U}}+\beta _{R_{U}})I_{U}-\beta _{E}E,( \beta _{R_{D}}+ \beta _{Ex})I_{D}-\beta _{I_{U}}I_{U}, \\ &\quad \frac{S_{M}}{N} (\alpha _{E}E+\alpha _{I_{U}}I_{U}+ \alpha _{I_{D}}I_{D}), \mu _{L}\frac{S_{L}}{N} ( \alpha _{E}E+\alpha _{I_{U}}I_{U} \\ & \quad {}+\alpha _{I_{D}}I_{D}) \biggr)^{T}. \end{aligned} \end{aligned}$$

Following the same arguments as those of Lemma 1 in van Driessche and Watmough [41], we have

$$\begin{aligned} D\mathscr{\widehat{F}}(\mathbf{\widehat{x}}) =& \begin{pmatrix} \widehat{F}&0 \\ 0&0 \end{pmatrix}, \end{aligned}$$

where

$$\begin{aligned} \widehat{F} =& \begin{pmatrix} \alpha _{E} \frac{\overline{S}_{M}+\mu _{L}\overline{S}_{L}}{N}& \alpha _{I_{U}} \frac{\overline{S}_{M}+\mu _{L}\overline{S}_{L}}{N}& \alpha _{I_{D}} \frac{\overline{S}_{M}+\mu _{L}\overline{S}_{L}}{N} \\ 0&0&0 \\ 0&0&0 \end{pmatrix}, \end{aligned}$$

and

$$\begin{aligned} D\mathscr{\widehat{V}}(\mathbf{\widehat{x}}) =& \begin{pmatrix} \widehat{V}&0 \\ J_{3}&0 \end{pmatrix}, \end{aligned}$$

where

18 $$\begin{aligned} \widehat{V}= \begin{pmatrix} \beta _{E}&0&0 \\ -\beta _{E}&\beta _{I_{U}}+\beta _{R_{U}}&0 \\ 0&-\beta _{I_{U}}&\beta _{R_{D}}+\beta _{Ex} \end{pmatrix}, \end{aligned}$$

and

$$ J_{3}= \begin{pmatrix} \alpha _{E} \frac{\overline{S}_{M}}{N}&\alpha _{I_{U}} \frac{\overline{S}_{M}}{N}&\alpha _{I_{D}} \frac{\overline{S}_{M}}{N} \\ \mu _{L}\alpha _{E} \frac{\overline{S}_{M}}{N}&\mu _{L}\alpha _{I_{U}} \frac{\overline{S}_{M}}{N}&\mu _{L}\alpha _{I_{D}} \frac{\overline{S}_{M}}{N} \end{pmatrix}. $$

It is clear that all diagonal elements of *V̂* are positive, whence *V̂* is a nonsingular *M*-matrix, see [35–39] and *F̂* is a nonnegative matrix. As in [2, 42], we refer to $\widehat{F}\widehat{V}^{-1}$ as the next generation matrix of the model equations (13)–(16) and we define the basic reproduction number $\mathscr{R}_{0}$ as follows:

19 $$\begin{aligned} \mathscr{R}_{0} = & \rho \bigl(\widehat{F}\widehat{V}^{-1} \bigr), \\ = & \biggl(\frac{\alpha _{E}}{\beta _{E}} + \frac{\alpha _{I_{U}}}{\beta _{I_{U}}+\beta _{R_{U}}} + \frac{\alpha _{I_{D}}\beta _{I_{U}}}{(\beta _{Ex}+\beta _{R_{D}})(\beta _{I_{U}}+\beta _{R_{U}})} \biggr) \frac{\overline{S}_{M}+\mu _{L}\overline{S}_{L}}{N} , \end{aligned}$$

where $\rho (A)$ denotes the spectral radius of the matrix *A* given in (17).

The free infected compartments including $S_{M}$, $S_{L}$ are monotonically decreasing, whereas the compartments of recovered and defunct individuals $R_{U}$, $R_{D}$, *Ex* are monotonically increasing and converge to their asymptotic equilibrium

$$ (\overline{S}_{M}, \overline{S}_{L}, \overline{R}_{U}, \overline{R}_{D},\overline{Ex} )^{T} $$

if and only if the compartments of infected individuals $(E,I_{U},I_{D} )^{T}$ converge to the zero equilibrium, taking into account the time varying of the compartments of susceptible individuals, and converge to the steady state value. Thus we have the following theorem about the asymptotic stability of (13)–(16). Now we are ready to state the main theorem concerning the local asymptotic stability of the endemic equilibrium.

### Theorem 3.4

*The endemic equilibrium* $\mathbf{X}^{*} = (0, 0,0, \overline{S}_{M}, \overline{S}_{L}, \overline{R}_{U}, \overline{R}_{D},\overline{Ex} )^{T}$ *of the model equations* (1)*–*(8) *exists and is locally asymptotically stable during each time episode of the lockdown interval* $[t_{i},t_{i+1}]$, *where* $(t_{i})_{0\leq i \leq n+1}$ *is an increasing positive sequence if and only if* $\mathscr{R}_{0}<1$, *where* $\mathscr{R}_{0}$ *is the basic reproduction number given in* (19). *In other words*, *if and only if*

$$\begin{aligned} \frac{\overline{S}_{M}+\mu _{L}\overline{S}_{L}}{N} < &\beta _{E} ( \beta _{I_{U}}+\beta _{R_{U}} ) \frac{\beta _{Ex}+\beta _{R_{D}}}{\mathcal{P}(\alpha ,\beta )}, \end{aligned}$$

*where*

$$\begin{aligned} \mathcal{P}(\alpha ,\beta ) =&\alpha _{E}(\beta _{Ex}\beta _{R_{U}}+ \beta _{R_{D}}\beta _{R_{U}}+\beta _{I_{U}} \beta _{Ex}+\beta _{I_{U}} \beta _{R_{D}}) \\ &{}+\beta _{E}(\alpha _{I_{U}}\beta _{Ex}+\beta _{I_{U}}\alpha _{I_{D}}+ \alpha _{I_{U}}\beta _{R_{D}}). \end{aligned}$$

### Proof

To study the local asymptotic stability of (1)–(8) around the equilibrium $\mathbf{X}^{*}$ in each time episode of the lockdown interval $[t_{i},t_{i+1}]$, we will focus first on the Jacobian subsystems matrices *F̂* and *V̂* including the compartments *E*, $I_{U}$, $I_{D}$, $S_{M}$, $S_{L}$ around the equilibrium $(0, 0,0, \overline{S}_{M}, \overline{S}_{L} )^{T}$ of the model equations (13)–(14). By using the inverse-positivity characterization of nonsingular *M*-matrices given in [39] and taking into account that *V̂*, given in (18), is a nonsingular *M*-matrix and

$$\begin{aligned} \widehat{F} =& \begin{pmatrix} \alpha _{E} \frac{\overline{S}_{M}+\mu _{L}\overline{S}_{L}}{N}& \alpha _{I_{U}} \frac{\overline{S}_{M}+\mu _{L}\overline{S}_{L}}{N}& \alpha _{I_{D}} \frac{\overline{S}_{M}+\mu _{L}\overline{S}_{L}}{N} \\ 0&0&0 \\ 0&0&0 \end{pmatrix} \end{aligned}$$

is nonnegative, it is readily concluded that $\widehat{F}\widehat{V}^{-1}$ is nonnegative. Let us consider the following matrix difference:

$$\begin{aligned} -J_{1} =&\widehat{V}-\widehat{F}, \\ =& \begin{pmatrix} \beta _{E}-\alpha _{E} \frac{\overline{S}_{M}+\mu _{L}\overline{S}_{L}}{N}&-\alpha _{I_{U}} \frac{\overline{S}_{M}+\mu _{L}\overline{S}_{L}}{N}&-\alpha _{I_{D}} \frac{\overline{S}_{M}+\mu _{L}\overline{S}_{L}}{N} \\ -\beta _{E}&\beta _{I_{U}}+\beta _{R_{U}}&0 \\ 0&-\beta _{I_{U}}&\beta _{R_{D}}+\beta _{Ex} \end{pmatrix}. \end{aligned}$$

It is clear that $-J_{1}$ and $-J_{1}\widehat{V}^{-1}=I-\widehat{F}\widehat{V}^{-1}$ have the $\mathbb{Z}$ sign pattern. Then, by using Lemma 3.3, we conclude that $-J_{1}$ is a nonsingular *M*-matrix if and only if $I-\widehat{F}\widehat{V}^{-1}$ is a nonsingular *M*-matrix. The spectral radius (*SR*) characterization of nonsingular *M*-matrices guarantees that $I-\widehat{F}\widehat{V}^{-1}$ is a nonsingular *M*-matrix if and only if $\rho (\widehat{F}\widehat{V}^{-1})<1$. Again, using (*SA*) and the basic reproduction number $\mathscr{R}_{0}$ given in (19), it follows that $\Re (\lambda _{i}(J_{1}))<0$ if and only if $\mathscr{R}_{0}=\rho (\widehat{F}\widehat{V}^{-1})<1$. □

Now, we can define the effective reproduction number $\mathscr{R}_{e}$ as the number of cases that one single infected individual is capable of infecting during different time episodes of the lockdown interval $[t_{i},t_{i+1}]$, $0\leq i \leq n$. In other words, we are going to protect uninfected individuals taking into account the efficiency of the control measures $C_{M}$ imposed by the authorities proposed in (9). Furthermore,

20 $$\begin{aligned} \mathscr{R}_{e}(t) = & C_{M}(t)\mathscr{R}_{0}, \end{aligned}$$

where $\mathscr{R}_{0}$ is the basic reproduction number given in (19).

## Data collection and parameter estimations

**Figure 3:**
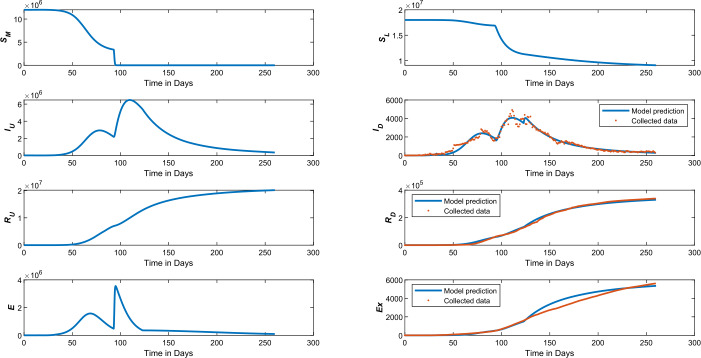
Model prediction taking into account different stages of the control measures imposed by the authorities and the corresponding epidemiological data, including the number of individuals diagnosed with (*COVID-19*), recovered and dead, collected from the Saudi Arabian Ministry of Health, see “https://covid19.moh.gov.sa/”

Looking at the response to the *COVID-19* spread in Saudi Arabia, we are going to present different stages of the actions taken by the authorities, taking into account the intensity of the restrictive measures in different time episodes. In the interest of simplicity and considering various control measures within the country, we will divide our time period in four different episodes, namely, from day 0 to day 30, from day 30 to day 90, from day 90 to day 120, and from day 120 to day 260. The data were collected from the Saudi Arabian Ministry of Health, see “https://covid19.moh.gov.sa/”, and present the number of diagnosed *COVID-19* infections, recovered and extinct individuals every day over a 9-month period. These data will be used to estimate the parameters over a given period of the duration of the restriction. It will be assumed that all parameters of our model are given in Table 1. We conduct a sensitivity analysis to determine which model parameters have a strong influence on the dynamics of the model equations (1)–(8), see [19, 20] for more details, on the parameter estimates methodology using differential equations. We are going to estimate the control parameters and the parameters of recovered and the dead compartments over different time episodes by using multi-objective optimization. We will perform several fits for each time episode using the Nelder–Mead search algorithm to find a local minimum. We set

$$\begin{aligned} \mathcal{J}\bigl(\mathbf{x}(\theta )\bigr) :=&\sum^{N}_{j=1} \bigl(x(t_{j},\theta )-d_{j}\bigr)^{2}, \end{aligned}$$

where $d_{j}$ represents the data measurement available at the time $t_{j}$, *N* stands for the number of measurements, and $\mathbf{x}(t_{j},\theta )$ represents the (*COVID-19*) model equations (1)–(8). The best fit relative estimation of the control parameters $\alpha _{E}$, $\alpha _{I_{U}}$, $\alpha _{I_{D}}$, $\beta _{I_{U}}$, $\beta _{E}$, and the estimated parameters $\beta _{R_{U}}$, $\beta _{R_{D}}$, $\beta _{Ex}$ of recovered, and the dead compartments are given in Table 2, with different time episodes. The model estimations compared to the given data of the number of diagnosed *COVID-19* infections, recovered and extinct individuals every day over a 9-month period are shown in Fig. 3.

**Table 2:** Estimated parameters of model (1)–(8), taking into account different control stage measures

Parameters	0 to 30 days	30 to 90 days	90 to 120 days	120 to 260 days.
$\alpha _{E}$	0.970103	0.745319	25.285168	60.624593.
$\alpha _{I_{U}}$	0.244387	0.000002	0.000688	0.000007.
$\alpha _{I_{D}}$	0.014381	0.000061	0.000091	0.000131.
$\beta _{E}$	0.1818	0.1818	0.190241	0.076462.
$\beta _{I_{U}}$	0.003330	0.000645	0.000464	0.000958.
$\beta _{R_{U}}$	0.0752	0.0752	0.0352	0.0352.
$\beta _{R_{D}}$	0.18554	0.779554	0.726325	1.1825.
$\beta _{Ex}$	0.010549	0.006583539	0.009839	0.02299.
$\mu _{L}$	0.05	0.05	0.01	0.00333333.

## Concluding remarks and suggestions

It is necessary to achieve a better understanding of the *COVID-19* dynamics, taking into account different control measures in order to reduce the number of infections and to decrease the mortality rate. The model proposed in the present manuscript has the advantage of describing the best way of controlling the pandemic. As was demonstrated in the parameter estimation, the ability to reduce the parameters $\alpha _{I_{U}}$, $\alpha _{I_{D}}$, and $\beta _{I_{U}}$ in this case will result in the detection of the number of infected individuals and in the reduction of the number of undiagnosed individuals by enhancing the testing. This will facilitate the control of the virus. Again, considering the effective reproduction number $\mathscr{R}_{e}(t)$ given in (20), it is clear that if we reduce the number of the more susceptible individuals and the parameter $\mu _{L}$ by respecting the social distancing and wearing masks, it will help with the control of the pandemic.

We aim at improving the model by including the number of individuals in intensive care and by adding a modification that allows for the effect of a vaccine, as given in [43] for measles epidemics. Another important effect that should be included will be the incubation time delays of receiving *COVID-19* positive test from the authority.

The study of persistence and stability in conjunction with the reproduction number is of paramount importance for the parameter estimation. We believe that this model provides a good basis for the study of more general cases, in which these parameters vary over each time interval. It will be of interest to develop a parallel algorithm, see [44, 45], in order to reduce the time needed to find the best fit of the multi-objective cost functional.

## Data Availability

All data generated or analyzed during this study are included in this work, see Fig. 3.

## References

[1] Ma Z., Li J. (2009). Dynamical Modeling and Analysis of Epidemics.

[2] Diekmann O., Heesterbeek H., Britton T. (2013). Mathematical Tools for Understanding Infectious Disease Dynamics.

[3] Ramos A.M., Ferrández M.R., Vela-Pérez M., Kubik A.B., Ivorra B. (2021). A simple but complex enough *θ*-SIR type model to be used with COVID-19 real data. Application to the case of Italy. Physica D.

[4] Enrique Amaro J., Dudouet J., Orce J.N. (2021). Global analysis of the COVID-19 pandemic using simple epidemiological models. Appl. Math. Model..

[5] Kudryashov N.A., Chmykhov M.A., Vigdorowitsch M. (2021). Analytical features of the SIR model and their applications to COVID-19. Appl. Math. Model..

[6] De la Sen M., Ibeas A. (2020). On a SIR epidemic model for the COVID-19 pandemic and the logistic equation. Discrete Dyn. Nat. Soc..

[7] Morato M.M., Bastos S.B., Cajueiro D.O., Normey-Rico J.E. (2020). An optimal predictive control strategy for COVID-19 (SARS-CoV-2) social distancing policies in Brazil. Annu. Rev. Control.

[8] Kermack W.O., McKendrick A.G. (1991). Contributions to the mathematical theory of epidemics–I. 1927. Bull. Math. Biol..

[9] Kermack W.O., McKendrick A.G. (1991). Contributions to the mathematical theory of epidemics–II. The problem of endemicity. 1932. Bull. Math. Biol..

[10] Kermack W.O., McKendrick A.G. (1991). Contributions to the mathematical theory of epidemics–III. Further studies of the problem of endemicity. 1933. Bull. Math. Biol..

[11] Li M.Y., Smith H.L., Wang L. (2001). Global dynamics an SEIR epidemic model with vertical transmission. SIAM J. Appl. Math..

[12] van den Driessche P., Watmough J. (2000). A simple SIS epidemic model with a backward bifurcation. J. Math. Biol..

[13] Liu X., Stechlinski P. (2017). Infectious Disease Modeling. A Hybrid System Approach.

[14] Bachar M., Dorfmayr A. (2004). HIV treatment models with time delay. C. R. Biol..

[15] Blackwood J.C., Childs L.M. (2018). An introduction to compartmental modeling for the budding infectious disease modeler. Lett. Biomath..

[16] Liu Z., Magal P., Seydi O., Webb G. (2020). Understanding unreported cases in the COVID-19 epidemic outbreak in Wuhan, China, and the importance of major public health interventions. Biology.

[17] Liu Z., Magal P., Seydi S., Glenn W. (2020). Predicting the cumulative number of cases for the COVID-19 epidemic in China from early data. Math. Biosci. Eng..

[18] Ivorra B., Ferrández M., Vela-Pérez M., Ramos A. (2020). Mathematical modeling of the spread of the coronavirus disease 2019 (COVID-19) taking into account the undetected infections. The case of China. Commun. Nonlinear Sci. Numer. Simul..

[19] Batzel J.J., Bachar M., Karemaker J.M., Kappel F. (2013). Merging mathematical and physiological knowledge: dimensions and challenges. Mathematical Modeling and Validation in Physiology.

[20] Batzel J.J., Bachar M. (2010). Modeling the cardiovascular-respiratory control system: data, model analysis, and parameter estimation. Acta Biotheor..

[21] Bachar M., Raimann J.G., Kotanko P. (2016). Impulsive mathematical modeling of ascorbic acid metabolism in healthy subjects. J. Theor. Biol..

[22] Lin Q., Zhao S., Gao D., Lou Y., Yang S., Musa S.S., Wang M.H., Cai Y., Wang W., Yang L., He D. (2020). A conceptual model for the coronavirus disease 2019 (COVID-19) outbreak in Wuhan, China with individual reaction and governmental action. Int. J. Infect. Dis..

[23] Chowell G., Fenimore P.W., Castillo-Garsow M.A., Castillo-Chavez C. (2003). SARS outbreaks in Ontario, Hong Kong and Singapore: the role of diagnosis and isolation as a control mechanism. J. Theor. Biol..

[24] Setti L., Passarini F., De Gennaro G., Barbieri P., Perrone M.G., Borelli M., Palmisani J., Di Gilio A., Piscitelli P., Miani A. (2020). Airborne transmission route of COVID-19: why 2 meters/6 feet of inter-personal distance could not be enough. Int. J. Environ. Res. Public Health.

[25] Liu Y., Ning Z., Chen Y., Guo M., Liu Y., Gali N.K., Sun L., Duan Y., Cai J., Westerdahl D., Liu X., Xu K., Ho K.-F., Kan H., Fu Q., Lan K. (2020). Aerodynamic analysis of SARS-CoV-2 in two Wuhan hospitals. Nature.

[26] Prather K.A., Wang C.C., Schooley R.T. (2020). Reducing transmission of SARS-CoV-2. Science.

[27] Leung N.H.L., Chu D.K.W., Shiu E.Y.C., Chan K.-H., McDevitt J.J., Hau B.J.P., Yen H.-L., Li Y., Ip D.K.M., Peiris J.S.M., Seto W.-H., Leung G.M., Milton D.K., Cowling B.J. (2020). Respiratory virus shedding in exhaled breath and efficacy of face masks. Nat. Med..

[28] Konda A., Prakash A., Moss G.A., Schmoldt M., Grant G.D., Guha S. (2020). Aerosol filtration efficiency of common fabrics used in respiratory cloth masks. ACS Nano.

[29] Tellier R., Li Y., Cowling B.J., Tang J.W. (2019). Recognition of aerosol transmission of infectious agents: a commentary. BMC Infect. Dis..

[30] Mittal R., Ni R., Seo J.-H. (2020). The flow physics of Covid-19. J. Fluid Mech..

[31] Buonanno G., Stabile L., Morawska L. (2020). Estimation of airborne viral emission: quanta emission rate of SARS-CoV-2 for infection risk assessment. Environ. Int..

[32] Li R., Pei S., Chen B., Song Y., Zhang T., Yang W., Shaman J. (2020). Substantial undocumented infection facilitates the rapid dissemination of novel coronavirus (SARS-CoV-2). Science.

[33] Meiss J.D. (2007). Differential Dynamical Systems.

[34] Hirsch W.M., Hanisch H., Gabriel J.-P. (1985). Differential equation models of some parasitic infections: methods for the study of asymptotic behavior. Commun. Pure Appl. Math..

[35] Dellacherie C., Martínez S., San Martín J. (2020). Inverse *M*-matrix, a new characterization. Linear Algebra Appl..

[36] Neumann M., Plemmons R.J. (1980). *M*-Matrix characterizations. II. General *M*-matrices. Linear Multilinear Algebra.

[37] Plemmons R.J. (1977). *M*-Matrix characterizations. I. Nonsingular *M*-matrices. Linear Algebra Appl..

[38] Poole G., Boullion T. (1974). A survey on *M*-matrices. SIAM Rev..

[39] Berman A., Plemmons R.J. (1994). Nonnegative Matrices in the Mathematical Sciences.

[40] Horn R.A., Johnson C.R. (1991). Topics in Matrix Analysis.

[41] van den Driessche P., Watmough J. (2002). Reproduction numbers and sub-threshold endemic equilibria for compartmental models of disease transmission. Math. Biosci..

[42] Diekmann O., Heesterbeek J.A.P., Metz J.A.J. (1990). On the definition and the computation of the basic reproduction ratio $R_{0}$ in models for infectious diseases in heterogeneous populations. J. Math. Biol..

[43] Stone L., Shulgin B., Agur Z. (2000). Theoretical examination of the pulse vaccination policy in the SIR epidemic model. Proceedings of the Conference on Dynamical Systems in Biology and Medicine.

[44] Gander M.J., Hairer E. (2008). Nonlinear convergence analysis for the parareal algorithm. Domain Decomposition Methods in Science and Engineering XVII.

[45] Gander M.J., Kulchytska-Ruchka I., Niyonzima I., Schöps S. (2019). A new parareal algorithm for problems with discontinuous sources. SIAM J. Sci. Comput..

